# Factors associated with fear of falling in people with Parkinson’s disease

**DOI:** 10.1186/1471-2377-14-19

**Published:** 2014-01-24

**Authors:** Beata Lindholm, Peter Hagell, Oskar Hansson, Maria H Nilsson

**Affiliations:** 1 Department of Neurology, Skåne University Hospital, Malmö, Sweden; 2 The PRO-CARE Group, School of Health and Society, Kristianstad University, Kristianstad, Sweden; 3 Department of Clinical Sciences, Lund University, Malmö, Sweden; 4 Department of Health Sciences, Lund University, Box 157, Lund SE-221 00, Sweden

**Keywords:** Fear of falling, Physical therapy, Parkinson’s disease, Postural Balance, Rehabilitation

## Abstract

**Background:**

This study aimed to comprehensibly investigate potential contributing factors to fear of falling (FOF) among people with idiopathic Parkinson’s disease (PD).

**Methods:**

The study included 104 people with PD. Mean (SD) age and PD-duration were 68 (9.4) and 5 (4.2) years, respectively, and the participants’ PD-symptoms were relatively mild. FOF (the dependent variable) was investigated with the Swedish version of the Falls Efficacy Scale, i.e. FES(S). The first multiple linear regression model replicated a previous study and independent variables targeted: walking difficulties in daily life; freezing of gait; dyskinesia; fatigue; need of help in daily activities; age; PD-duration; history of falls/near falls and pain*.* Model II included also the following clinically assessed variables: motor symptoms, cognitive functions, gait speed, dual-task difficulties and functional balance performance as well as reactive postural responses.

**Results:**

Both regression models showed that the strongest contributing factor to FOF was walking difficulties, i.e. explaining 60% and 64% of the variance in FOF-scores, respectively. Other significant independent variables in both models were needing help from others in daily activities and fatigue. Functional balance was the only clinical variable contributing additional significant information to model I, increasing the explained variance from 66% to 73%.

**Conclusions:**

The results imply that one should primarily target walking difficulties in daily life in order to reduce FOF in people mildly affected by PD. This finding applies even when considering a broad variety of aspects not previously considered in PD-studies targeting FOF. Functional balance performance, dependence in daily activities, and fatigue were also independently associated with FOF, but to a lesser extent. Longitudinal studies are warranted to gain an increased understanding of predictors of FOF in PD and who is at risk of developing a FOF.

## Background

Approximately 75% of people with Parkinson’s disease (PD) have an impaired balance [1], which constitutes one of the most distressing symptoms [2]. People with PD are particularly unstable when perturbed backwards due to impaired postural reflexes [3-5]*,* which is suggested to be evaluated clinically by using an unexpected shoulder pull [6]. Already early during the disease, turning difficulties are common [7] and an unsteadiness while turning is also associated with having more severe freezing of gait (FOG) [8]. Walking difficulties are also common and mainly characterized by a decreased gait speed and shuffling gait. Gait and balance problems are also related to non-motor features (e.g. cognitive dysfunction) of PD and are exacerbated by dual tasking [9].

People with PD have an increased risk of falling as compared to healthy individuals at the same age, but also in relation to people with other neurological disorders [10-12]. They usually fall while performing activities such as walking, turning, transferring to/from sitting, bending forwards or while reaching [13]. It is also common for people with PD to experience near falls, which can be defined as “a fall initiated but arrested by support from a wall, railing, other person, etc.” [14]. A recent review scrutinized specific factors associated with recurrent falls among people with PD, and fear of falling (FOF) was then highlighted as one of the risk factors [15]. In addition, FOF has been shown to be a predictor for community walking [16] and a major barrier to engaging in exercise [17]. FOF can be defined as a lack of confidence (low self-efficacy [18]) to be able to perform activities without falling, i.e. low fall-related self-efficacy.

Among people with PD, FOF is common and about 70% report activity limitations due to FOF, which also may cause social isolation [10,19]. Although FOF influences activity and participation negatively among people with PD, there is yet limited knowledge regarding contributing factors. Such knowledge is highly warranted in order to develop means that efficiently tap causal factors. At present, there are four published studies that used multivariate analysis to investigate contributing factors to FOF in PD [1,20-22]*.* Two out of these four studies were postal surveys and lacked clinical data [1,21], and none of them have been replicated [1,20-22]. More importantly, no study has included independent variables targeting functional balance performance, dual tasking, and gait speed or used an unexpected shoulder pull when assessing postural instability. Since gait speed and functional balance performance have been shown to correlate to FOF in bivariate analyses [23,24], these aspects may tentatively be of importance when investigating contributing factors to FOF. Dual-tasking might also be of interest since it worsens gait impairments in PD and may lead to wrong prioritization, i.e. the “posture second” strategy [9,25]. There is thus a need for a more thorough understanding of contributing factors to FOF in PD in order to address this efficiently in clinical practice and research.

This study aimed at determining factors associated with FOF (conceptualized as low fall-related self-efficacy) among people with PD. More specifically, the aim was to determine whether previous postal survey based findings could be replicated in an independent clinical sample and, secondly to investigate whether additional and previously unexplored motor aspects (e.g. gait speed, functional balance performance) as well as cognitive features independently may contribute to FOF.

## Methods

All people diagnosed with PD receiving care at a south Swedish university hospital during 2007–2011 were considered eligible for inclusion (n = 273). Exclusion criteria were age above 80 years old (n = 106), inability to stand without support (n = 17), inability to understand instructions (n = 8) or being mentally or medically unstable (n = 7). The remaining 135 patients were invited to participate. Twenty-eight (12 women) participants declined to participate, and they did not differ significantly (p ≥ 0.07, the Mann–Whitney U test) from the included ones with respect to age and PD-duration. Three additional participants (2 women) were excluded due to missing data on the dependent variable: the Swedish version of the Falls Efficacy Scale, i.e. FES(S). The final study sample consisted of 104 participants.

### Ethics statement

The Regional Ethical Review Board in Lund (Sweden) approved the study (Dnr 2011/768). All participants gave written informed consent.

#### Instruments

Demographic questions included, e.g., age, sex and disease duration. Additional questions (no/yes responses) included experience of falls during the past six months [26], near falls [14], dual-task difficulties (*“Do you experience balance problems when doing more than one thing at a time, e.g. carrying a tray while walking?”*) and pain (*“Do you presently suffer from pain?”*). For descriptive purposes, an additional dichotomous question (no/yes) specifically targeted FOF.

A battery of self-reported questionnaires was included. FES(S) targets fall-related self-efficacy, and includes 13 items (activities) rated from 0 (not confident at all) to 10 (completely confident) [23,27]. The maximum total score is 130 and higher scores denote “better” balance confidence. The self-administered version [8] of the freezing of gait questionnaire (FOGQsa) [28] consists of six items scored 0–4 (higher scores = more difficulties). In this study, we only used items 3 (freezing) and 6 (turning hesitations). Those scoring *≥*1 on item 3 were categorized as “freezers” and those scoring *≥*1 on item 6 were considered as having turning hesitations [1]. The generic Walk-12 (Walk-12G) assesses walking difficulties in everyday life, and the total score ranges from 0 to 42 (higher scores = more walking difficulties) [29]. The Functional Assessment of Chronic Illness Therapy - Fatigue scale (FACIT-F) consists of 13 items with a total score ranging from 0 to 52 (higher scores = less fatigue) [30,31]. The Parkinson's disease Activities of Daily Living Scale (PADLS) is a five-grade (5 = worse) single-item scale regarding ADL-difficulties [32,33]. Those scoring >2 were categorized as “needing help from others in daily activities”.

Before clinical assessments, all participants self-rated their motor status at the time of examination as “good/on”, “on with dyskinesias”, or “bad/off”. Clinical assessments targeted functional balance, retropulsion due to abnormal reactive postural responses, gait speed, parkinsonian motor status and cognition. The Berg balance scale (BBS) was used to assess functional balance performance of importance in daily life [34]. It includes 14 items (tasks) scored 0–4, and the maximum score is 56 (56 = better) [34,35]. The Nutt retropulsion test (NRT) assesses reactive postural responses [6,36]. The patient then stands with eyes open and feet slightly apart; the examiner stands behind the patient and gives (without prior warning) a sudden, firm and quick backward pull to the shoulders. Only one trial was performed (scored 0–3, 3 = worse) [6], and those scoring ≥1 were categorized as having abnormal reactive postural responses. The 10-meter walk test (10MWT) was used to measure gait speed [35]. It was performed in both comfortable and fast walking speed (randomized order, two trials each). In this study, we only used comfortable gait speed and a total distance of 14 meters, from which gait speed (m/s) was calculated for the mid 10 meters. The trial with the highest comfortable gait speed was used in the analyses. Parkinsonian motor symptoms were assessed with the Unified PD Rating Scale (UPDRS) part III (motor examination) [35,37]. It consists of 14 items (graded 0–4) with a total score ranging from 0 to 108 (108 = worse). In addition, dyskinesia was self-rated using part IV (complications of therapy) of the UPDRS; those scoring ≥1 on item 32 (dyskinesia duration) were categorized as having dyskinesias [37]. The Mini-Mental State Examination (MMSE) was used as a coarse cognitive test [38], and yields a total score ranging between 0–30 (30 = better).

#### Procedure

All participants were assessed during an outpatient visit, which was scheduled at a time of day when the participant usually reported to feel at best. First, the participants completed the self-administered questionnaire booklet. Thereafter, all participants were evaluated by the same physical therapist (BL). Clinical assessments were performed in the following order: BBS; NRT; 10 MWT; UPDRS part III; and the MMSE. These were followed by additional self-administered questions targeting dyskinesia and demographic information.

#### Statistical analyses

Data were checked regarding underlying assumptions and described and analyzed accordingly using IBM SPSS version 19. The alpha level of significance was set at 0.05 (2-tailed, exact *P*-values were used). Spearman correlations (*r*_*s*_) and Mann–Whitney *U*-tests were used for bivariate analyses of associations with the dependent variable FES(S). Forward multiple linear regression models were used based on the results from a recently published study [1]. In our first model, we replicated the model identified by Nilsson et al. [1] by using age, disease duration, walking difficulties, fatigue, need help from others in daily activities, turning hesitations, freezing of gait, dyskinesia, experiencing falls or near falls, and pain as independent variables. In our second model, we explored the effects of taking dual*-*task difficulties and variables based on clinical examination, i.e., parkinsonian motor symptoms (UPDRS III), cognition (MMSE), balance (NRT, BBS) and gait speed (10MWT) into account as additional independent variables. Models were checked regarding underpinning assumptions.

## Results

Sample characteristics and results from bivariate analyses are presented in Table 1. According to the dichotomous FOF-question, 38 out of 104 (37%) participants reported having FOF. FES(S) scores demonstrated significant bivariate associations with all variables but gender. The median FES(S) score was 117 (q1-q3, 69.5-129; min-max, 11–130). At the time of assessments, 91 out of the 104 participants (87.5%) rated their motor status as “on”, whereas 9 (8.7%) rated it as “on with dyskinesias”, and four (3.8%) rated it as “off”.

**Table 1:** Sample characteristics and bivariate associations with FES(S) scores

	**Total sample (n = 104)**		**Spearman correlations with FES(S) scores**		**P-value**
Age (years), mean (SD)	68 (9.4)		−0.270		0.006
PD-duration (years), mean (SD)	5 (4.2)		−0.350		&0.001
Cognition (MMSE), median (q1-q3)	28 (26–29)		0.220		0.027
Motor symptoms (UPDRS III), median (q1-q3)	13 (8–20)		−0.510		&0.001
Balance (BBS), median (q1-q3)	52.5 (46–55)		0.650		&0.001
Gait speed (10MWT) (m/s), median (q1-q3)	1.18 (0.95–1.35)		0.480		&0.001
Walking difficulties (Walk-12G), median (q1-q3)	8 (4.5–21)		−0.760		&0.001
Fatigue (FACIT-F), median (q1-q3)	38 (29–44)		0.710		&0.001
	**n (%)**^**a**^		**Median (q1-q3) FES(S) scores**^**a**^		**P-value Mann Whitney U-test**
	**No**	**Yes**	**No**	**Yes**	
Freezing of gait (item 3, FOGQsa)^b^	60 (58)	44 (42)	128 (112–130)	87 (44–117)	&0.001
Turning hesitations (item 6, FOGQsa)^c^	68 (65)	36 (35)	126 (105–130)	81 (39–113)	&0.001
Dyskinesias (item 32, UPDRS IV)^d^	66 (63)	38 (37)	124 (95–129)	101 (48–125)	0.009
Need help from others in daily activities (PADLS)^e^	93 (90)	11 (10)	122 (94–129)	33 (18–50)	&0.001
Experienced falls	76 (73)	28 (27)	124 (96–130)	89 (41–114)	&0.001
Experienced near falls	64 (62)	39 (38)	127 (106–130)	91 (43–116)	&0.001
Experienced balance problems while dual-tasking	52 (50)	52 (50)	128 (111–130)	94 (51–118)	&0.001
Pain	78 (75)	26 (25)	123 (94–130)	91 (43–124)	0.005
Retropulsion (NRT)^f^	78 (75)	26 (25)	124 (83–130)	104 (59–120)	0.011
Female gender	55 (53)	49 (47)	118 (87–129)	113 (61–129)	0.258

^a^Refers to the dichotomous (No/Yes) variables, and n (%) clarifies the number (percentage) of participants that either have or do not have the specified characteristic.

^b^Item 3 (“freezing”) of the FOGQsa. Those scoring ≥1 were categorized as freezers.

^c^Item 6 (“turning hesitations”) of the FOGQsa. Those scoring ≥1 were categorized as having turning hesitations.

^d^Item 32 of the UPDRS part IV. Those scoring ≥1 were categorized as having dyskinesias.

^e^Those scoring >2 on the PADLS were categorized as needing help from others in daily activities.

^f^Scores ≥1 on the NRT were categorized as having retropulsion.

BBS, Berg Balance Scale (possible scores, 0–56; higher = better); FACIT-F, the Functional Assessment of Chronic Illness Therapy - Fatigue scale (possible score, 0–52; higher = better); FES(S), Falls Efficacy Scale, Swedish version (possible scores, 0–130; higher = better); FOGQsa, Freezing of Gait Questionnaire, self-administered version; MMSE, Mini Mental State Examination (possible scores, 0–30; higher = better); NRT, Nutt Retropulsion Test (possible scores, 0–3; higher = worse); PADLS, the Parkinson’s disease Activities of Daily Living Scale (possible scores 1–5; higher = worse); PD, Parkinson’s disease; q1-q3, 1^st^-3^rd^ quartile; SD, standard deviation; UPDRS III, part III (motor score) of the Unified PD Rating Scale (possible scores, 0–108; higher = worse); UPDRS part IV (complications of therapy), item 32 (possible scores 0–4; higher = worse); 10MWT, 10-meter walking test; m/s, meters per second; Walk-12G, 12-item generic walking scale (possible scores, 0–42; higher = worse).

One participant had a missing value for the MMSE, and another participant had a missing value in relation to near falls.

The first multiple linear regression based on the results from Nilsson et al. [1] resulted in three significant independent variables explaining 66% of variance in FES(S) scores (Table 2). The strongest independent variable (as assessed by the standardized regression coefficients, *β*) was walking difficulties (Walk-12G scores), which could account for 59.5% of the variance in FES(S) scores. This was followed by fatigue and needing help from others in daily activities (Table 2).

**Table 2:** **Model I (replication**[1]**): multiple linear regression with fear of falling (FES(S) scores) as the dependent variable in people with Parkinson’s disease, n = 104**^**a**^

					**Adjusted R**^**2**^	
**Significant independent variables**^**b**^	**B (95% CI)**		***β***	**P-value**	**Stepwise change**	**Cumulative**
Walking difficulties (Walk-12G)	−1.844	(−2.423, −1.266)	−0.524	0.000	0.595	0.595
Need help from others in daily activities (PADLS)	−24.960	(−40.672, −9.247)	−0.213	0.002	0.042	0.637
Fatigue (FACIT-F)	0.667	(0.165, 1.169)	0.214	0.010	0.021	0.658

^a^Independent variables in the analysis were: need help from others in daily activities (PADLS: dichotomized, 1 = yes), walking difficulties (Walk-12G), fatigue (FACIT-F), age (years), PD-duration (years), falls (1 = yes), near falls (1 = yes), dyskinesia (dichotomized, 1 = yes), freezing (FOGQsa item 3: dichotomized, 1 = freezing), turning hesitations (FOGQsa item 6: dichotomized, 1 = turning hesitations), pain (dichotomized, 1 = yes).

^b^Listed by order of entry into the model (forward method).

FACIT-F, the Functional Assessment of Chronic Illness Therapy-Fatigue scale (0–52; higher = better); FES(S), Falls Efficacy Scale (0–130; higher = better); FOGQsa, Freezing of Gait Questionnaire, self-administered version (items are scored 0–4; higher = worse); PADLS, The Parkinson’s disease Activities of Daily Living Scale (1–5; higher = worse; those scoring >2 were categorized as needing help from others in daily activities) Walk-12G, 12-item generic walking scale (0–42; higher = worse).

B: regression coefficient; CI: confidence interval; *β*: standardized regression coefficient.

Adding information about the occurrence of dual-task difficulties and clinical assessments as independent variables resulted in a model with four independent variables explaining 73% of variance in FES(S) scores (Table 3). The three variables identified in the first model remained significant also in the second model, and the only variable that contributed additional explanatory power was functional balance (BBS). The strongest independent variable was still walking difficulties, followed by functional balance, needing help from others in daily activities and fatigue (Table 3).

**Table 3:** **Model II (extended): multiple linear regression with FES(S) scores as the dependent variable in people with Parkinson’s disease, n = 104**^**a**^

					**Adjusted R**^**2**^	
**Significant independent variables**^**b**^	**B (95% CI)**		***β***	**P-value**	**Stepwise change**	**Cumulative**
Walking difficulties (Walk-12G)	−1.543	(−2.118, −0.968)	−0.446	0.000	0.642	0.642
Need help from others in daily activities (PADLS)	−21.823	(−35.841, −7.806)	−0.189	0.003	0.045	0.687
Functional balance (BBS)	0.877	(0.333, 1.422)	0.221	0.002	0.027	0.714
Fatigue (FACIT-F)	0.547	(0.103, 0.991)	0.179	0.016	0.014	0.728

^a^Independent variables in the analysis were: need help from others in daily activities (PADLS: dichotomized, 1 = yes), walking difficulties (Walk-12G), fatigue (FACIT-F), age (years), PD-duration (years), falls (1 = yes), near falls (1 = yes), dyskinesia (item 32 UPDRS part IV: dichotomized, 1 = yes), freezing (FOGQsa item 3: dichotomized, 1 = freezing), turning hesitations (FOGQsa item 6: dichotomized, 1 = turning hesitations), pain (dichotomized, 1 = yes), cognition (MMSE), motor symptoms (UPDRS III), Balance (BBS), 10-meters walk test (comfortable gait speed), Nutt Retropulsion test (dichotomized, 1 = abnormal reactive postural response), self-reported dual-task difficulties (dichotomized, 1 = yes).

^b^Listed by order of entry into the model (forward method).

BBS, Berg balance scale, 0–56 (higher = better); FACIT-F, the Functional Assessment of Chronic Illness Therapy-Fatigue scale (0–52; higher = better); FES(S), Falls Efficacy Scale (0–130; higher = better); FOGQsa, Freezing of Gait Questionnaire, self-administered version (items are scored 0–4; higher = worse); MMSE, the Mini-Mental State Examination (possible scores, 0–30; higher = better); PADLS, the Parkinson’s disease Activities of Daily Living Scale (1–5; higher = worse; those scoring >2 were categorized as needing help from others in daily activities); PD, Parkinson’s disease; Walk-12G, 12-item generic walking scale (0–42; higher = worse); UPDRS III: motor part of the Unified PD Rating Scale; UPDRS IV: motor complications.

B: regression coefficient; CI: confidence interval; *β*: standardized regression coefficient.

## Discussion

By comprehensibly investigating contributing factors to FOF among people with PD and by using multivariate analyses, this study confirms previous observations suggesting that walking difficulties in daily life is the strongest contributing factor in addition to independence in daily activities and fatigue. Although some previous PD-studies have shown similar results [1,20], none included independent variables that targeted functional balance performance, dual-task difficulties, and gait speed. A novel finding in this study is that functional balance (that is of importance in daily activities) was identified as an additional significant independent contributor to FOF, whereas a reactive postural response after an external perturbation (and other motor or cognitive aspects) was not. Including functional balance performance in the model increased the explanatory power from 66% to 73%, whereas other motor and cognition aspects do not appear to provide any improvements beyond the first model. The present findings may have important implications for physical therapy and rehabilitation targeting PD.

Several variables that showed highly significant bivariate relationships with FOF (e.g. cognition and falls) were not independently associated with FOF when controlling for other independent variables. This illustrates a major pitfall in relying on bivariate analyses and highlights the importance of using multivariate analyses in this type of studies. Although it may appear surprising that falls did not contribute to FOF, this finding is in line with other PD-studies using multivariate analyses [1,20,22].

Our first regression model represents an independent replication of a prior study based on self-reported postal survey data [1]. The replication corroborates walking difficulties as a major contributing factor to low fall-related self-efficacy. This implies that walking difficulties should be a primary target when attempting to reduce FOF.

Although generally confirming previous findings, the present study did not identify turning hesitations as an independent contributor to FOF as shown in the study by Nilsson et al. [1]. This discrepancy is probably not related to differences in the dependent variable (i.e. FOF, operationalized as low fall-related self-efficacy), since the present median FES(S) score was similar to the one obtained in the study by Nilsson et al. (117 and 114, respectively) [1]. However, sample differences may still have contributed, as the present sample seemed to be less affected by their PD than the previous sample, e.g. proportions of fallers (33% versus 45% in the study by Nilsson et al. [1]) and of people needing help in daily activities (10% here versus 27%). An alternative explanation for the discrepancy may be that all independent variables were not identically assessed in the two studies.

Walking difficulties in daily life was identified as a major explanatory variable in both models, accounting for almost two thirds of the variance in FES(S) scores. This suggests that walking ability may be a primary therapeutic target for alleviating FOF. Functional balance performance (BBS scores) was significantly associated with FOF, whereas the NRT was not. The clinical implication of this finding is that balance training probably should focus on challenges induced by self-generated perturbations rather than external perturbations, if aiming at reducing FOF. In other words, it seems like interventions should target functional balance performance and not reactive postural responses if aiming at reducing FOF among people with mild PD.

FOF among people with PD needs specific attention since it has been identified as a risk factor for recurrent falls [15], a barrier for exercise [17], and a predictor for community walking [16]. Furthermore, FOF causes activity restrictions and avoidance as well as social isolation [10,19,23]. A recent Cochrane review concluded that physical therapy can yield short-term improvements in walking, mobility and balance as compared with no intervention in people with PD [39]. However, the review did not support reduction of FOF by physical therapy. This may be explained by several factors. For example, few of the reviewed studies included FOF as an outcome; compromised methodological quality of the included studies; or that the key ingredients of the interventions did not address walking difficulties in daily life. Future trials targeting walking ability and including FOF as an outcome are thus needed. Importantly, FOF may be such a complex construct that it best benefits from using an interdisciplinary approach. The latter may be supported by the fact that dependence in daily activities as well as fatigue was independently associated with FOF. Interestingly, it has been suggested that poor walking economy among people with PD may contribute to fatigue [40]. However, the exact role of this enigmatic complaint remains speculative [41-43] and cannot be addressed based on the current study.

### Limitations and future perspectives

This sample consisted of people with PD that were relatively mildly affected by their disease, which is mirrored by several of the descriptive variables, e.g. motor symptoms (UPDRS III), PD duration, gait speed, and the number of participants that had experienced falls. In addition, people being above the age of 80 years were not included. Our findings may thus not apply to very old people with PD or those with more severe PD. It should also be acknowledged that although several independent variables were included, there may be additional variables of importance for FOF such as general self-efficacy, environmental factors, anxiety and depression. In fact, a previous study that used multivariate analyses showed that greater depression contributed to perceived consequences of falling while anxiety contributed to activity avoidance due to the risk of falling [21]. However, ADL-difficulties showed a stronger independent association with activity avoidance than anxiety did. It should be noted that the study included few independent variables (disease severity, ADL, depression and anxiety), and the influence of anxiety and depression on FOF remains unclear due to the cross-sectional design of the study.

In the present study, some of the variables that did not show independent associations with FOF were assessed by relatively coarse indicators, e.g. dual-task difficulties and cognition (MMSE). By using a coarse indicator one may not capture those having mild problems. For instance, it has been suggested that the Montreal Cognitive Assessment (MoCA) is preferably to MMSE when screening for early cognitive impairments in PD [44,45]. Finally, due to the cross-sectional design of this study, it cannot be establish whether the identified associated factors actually are predictive of FOF. Longitudinal studies are needed to gain an increased understanding of risk factors for developing FOF, but also for determining factors that may aggravate existing FOF over time. Such knowledge is imperative to maximize the potential of interventions aiming at reducing FOF.

## Conclusions

This study was able to replicate previous main findings in an independent sample of people with PD by identifying everyday walking difficulties as a primary FOF associated factor, and additional independent contributions by fatigue and the need for help in daily activities. Furthermore, functional balance performance was found to be the only factor among a range of additional clinical motor and cognitive variables that was able to account for additional significant proportions of the variance in FOF. These observations imply that walking difficulties and balance performance in daily life are candidate therapeutic targets in order to reduce FOF in PD. However, longitudinal studies are warranted in order to gain an increased understanding of predictors of FOF in PD and who is at risk of developing a FOF.

## Competing interests

The authors have declared that no competing interests exist.

## Authors' contributions

BL, PH, OH and MHN conceived and designed the study. BL performed data collection. BL, MHN and PH analyzed the data. BL and MHN drafted the initial manuscript. All authors participated in writing (and approved) the final version of the manuscript.

## Pre-publication history

The pre-publication history for this paper can be accessed here:

http://www.biomedcentral.com/1471-2377/14/19/prepub
